# The effect of bundle care on central line associated bloodstream infections in two medical ICUS

**DOI:** 10.1186/2047-2994-4-S1-P211

**Published:** 2015-06-16

**Authors:** Y-W Sung, C-T Hung, M-J Huang, S-H Chen, J-R Tsai, Y-H Chen

**Affiliations:** 1 Department of Nursing, Kaohsiung Medical University Hospital(KMUH), Kaohsiung Medical University, Kaohsiung, Taiwan, Province of China; 2 Department of Infection Control Room, Kaohsiung Medical University Hospital(KMUH), Kaohsiung Medical University, Kaohsiung, Taiwan, Province of China; 3 Department of Internal Medicine and Intensive Care Unit, Kaohsiung Medical University Hospital(KMUH), Kaohsiung Medical University, Kaohsiung, Taiwan, Province of China; 4 Department of Internal Medicine, Kaohsiung Medical University Hospital(KMUH), Kaohsiung Medical University, Kaohsiung, Taiwan, Province of China; 5 Division of Infectious Diseases, Kaohsiung Medical University Hospital(KMUH), Kaohsiung Medical University, Kaohsiung, Taiwan, Province of China

## Introduction

Central lines are useful medical devices commonly used in the intensive care units (ICUs), whereas central line associated bloodstream infection (CLABSI) is also a common nosocomial infection in the ICUs. CLABSI may increase the use of antibiotics, hospital days, medical costs, and even the mortality rate. Therefore, developing and implementing adequate infection control measures to prevent CLABSI is always an important clinical question.

## Objectives

In this study, we investigated the effect of implementing bundle care for bloodstream infection (BC-BSI) in medical ICUs on reducing CLABSI.

## Methods

This study was conducted in two medical ICUs in a medical center in southern Taiwan from March, 2010 to December, 2014. These two units started implementation of BC-BSI on September, 2011. The BC-BSI included: (1) hand washing, (2) maximal barrier precautions (from head to toe), (3) skin preparation with 2% chlorhexidine, (4) avoidance of femoral insertion sites, (5) changing dressing every 48 hours (gauze dressing) or 7 days (transparent dressing), (6) daily review of the necessity of lines with prompt removal of unnecessary lines. We compared the rate of CLABSI and the use of central lines before and after the implementation of BC-BSI.

## Results

After the implementation of BC-BSI, the rate of CLABSI decreased from 5.45‰ to 4.25‰ and the use of central lines decreased from 59.60% to 55.55%

## Conclusion

The implementation of BC-BSI effectively decreased the use of central lines and the rate of CLABSI in medical ICUs. With the target of zero-tolerance to nosocomial infection, BC-BSI should be promoted and implemented in daily clinical practice to improve the quality of critical care.

## Disclosure of interest

None declared.

